# Fast preparation and recycling method for colloidal probe cantilevers in hydrophobic mapping applications

**DOI:** 10.1016/j.mex.2019.03.010

**Published:** 2019-03-18

**Authors:** B. Babel, M. Rudolph

**Affiliations:** Helmholtz Institute Freiberg for Resource Technology, Helmholtz-Zentrum Dresden-Rossendorf, 09599 Freiberg, Germany

**Keywords:** Colloidal probe preparation, Atomic force microscopy, Recyclable cantilevers, Fast colloidal probe preparation, Adhesion mapping, Contaminations

## Abstract

Probe contamination of atomic force microscope (AFM) tips with colloidal probes is limiting the lifetime of the probe and the reproducibility in force interaction measurements, rendering cantilevers useless. Earlier proposed cleaning methods like mechanical scrubbing, UV, plasma and solvent cleaning procedures have limitations especially for inorganic particulate contaminations. In this paper we demonstrate a fast procedure to recycle contaminated colloidal probe cantilevers and reequip them with pristine colloids without affecting the mechanical and structural properties of the cantilever. The proposed procedure reduces the total time for probe preparation and allows extended experimental test work with singular cantilevers reducing the deviations by cantilever calibration.

• fast preparation
• recyclable cantilevers

## Graphical abstract

**Figure:**
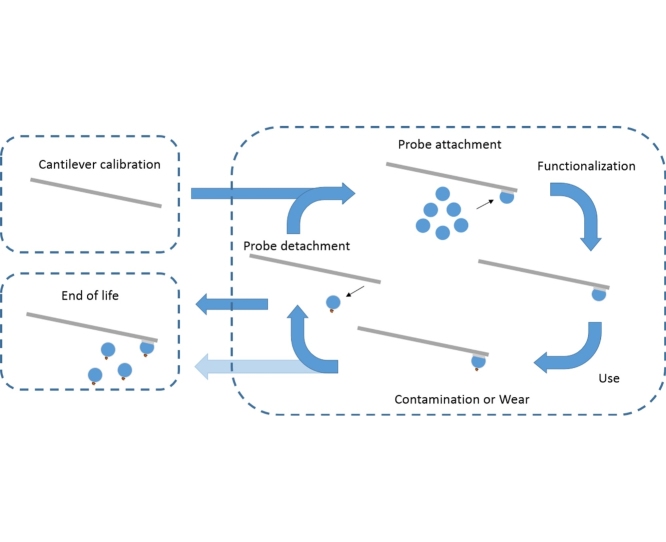

**Specifications Table**

**Table:** 

**Subject Area:**	*Chemical Engineering*
**More specific subject area:**	*Atomic force microscopy*
**Method name:**	*Colloidal probe preparation*
**Name and reference of original method:**	*The method was derived from multiple methods and cannot be associated to a singular reference.*
**Resource availability:**	*All reagents and instruments indicated are commercially available. The sources of specific components were indicated in the manuscript.*

## Method details

**Fig. 1:**
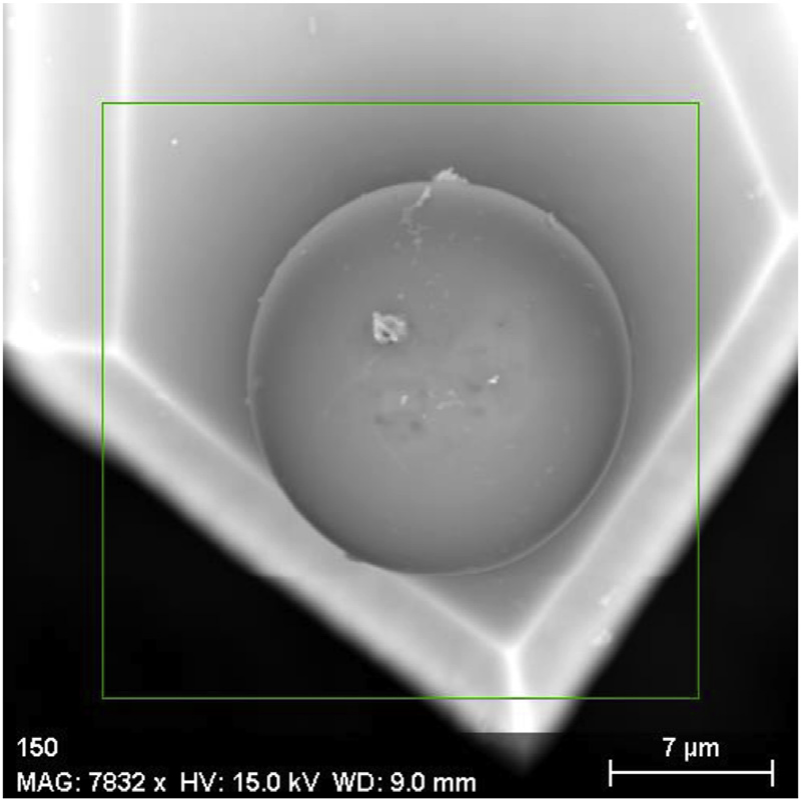
Scanning electron microscope (SEM) image of a colloidal probe prepared with this protocol after surface contamination during scanning. Fixating glue is not present on the upper half of the sphere.

Colloidal probe atomic force microscopy (CP-AFM) was first reported by [1,2] and is nowadays used in multiple applications in various fields of research. One possible application are adhesive force mappings on a variety of samples including biological [[3], [4], [5]], mineral [6,7], ceramic [[8], [9], [10]] or wooden samples [3,11]. Beside possible sources of contamination during storing in ambient conditions as summarized by [12], the main source of colloidal probe contamination in an adhesive force mapping application on mineral surfaces experienced by the authors are inorganic particulate contaminations [13], as given in Fig. 1. The contaminations originate from the sample preparation procedure either by constituents of the polishing suspension for the substrate, like abrasives and additives or the minerals themselves. In literature there are no reports on probe durability, reproducibility between different probes and or cantilevers in adhesion mapping applications. Wallqvist et al. reported on investigations with about 50 points [14] to 560 points [15] and an example with an extensive mapping (400 points) was reported by Xie et al. [16] with no comment on probe durability or reproducibility of measurements.

As the mechanical scrubbing procedure proposed by [12] could not be successfully applied and high loads possibly damage the functionalization of the colloid, we propose a fast probe preparation procedure for recyclable colloidal probe cantilevers. This includes the demonstration of a fast surface modification and probe removal method without the aid of solvents or acids as they possibly contain and transport contaminants, which are potentially problematic for the reflex coating of the cantilever. Additionally, this technique allows the use of interchangeable particles for example with different geometries for investigations with the same cantilever, reducing the deviations caused by cantilever calibration reported to be in the 10% range for resonance frequency calibration [17].

A variety of methods for cantilever or colloidal probe functionalization have been reported in literature, the same applies for possible colloids attached to the cantilever like glass spheres [18], polystyrene particles [6,7,19], silica microspheres [14,19,20] Al_2_O_3_ particles [[8], [9], [10]] or gold particles [14]. A broader overview of the colloidal probe method was compiled by [18], listing more possible probes like TiO_2_, MgO, ZnS, poly-(methyl methacrylate) and polyethylene. The functionality of the probe relies on functionalization or on natural surface properties e.g. polystyrene. For the hydrophobization of probes two mayor ways are reported. The first is an additional gold coating [16,21] or the use of a gold colloid [14] and the utilization of various thiols forming self-assembled monolayers (SAMs). The other method is the utilization of organosilanes in combination with hydroxylated surfaces, mostly silica spheres. Used reagents are alkyl or fluoroalkyl silanes in solvent or vapor phase deposition [[8], [9], [10],^14^,^20^,^22^].

Some authors consider gluing particles to cantilevers unacceptable due to surface contamination by the glue or solvents and suggested melting or sintering [18]. As this process is not reversible under standard lab conditions, this paper focusses on the utilization of glues for probe fixation. Previously reported glues are Norland Optical UV-adhesive NOA63 [23], Araldit® Rapid glue [14], Epikote 1004 [15] or other types of epoxy glues [6,7]. The application of UV glue seems favorable in terms of glue handling and reduction of curing times, both does not apply to rapid glues or epoxy based glues with a problematic handling or extensive times for curing respectively. In the following, this paper focuses on reported functionalization and preparation methods for hydrophobic colloidal probes, while the general concept of this paper can be applied to other surface functionalization methods. In general, the main steps in probe preparation are cantilever calibration depending on the method, particle cleaning, particle attachment, glue setting and the actual surface modification procedure. Rarely reported times for surface modification are ranging from overnight [14,16], 12 h–18 h [21], 20 h [14,15] up to 24 h [23]. With our suggested procedure, the time for glue setting and surface modification can be substantially reduced.

The adsorption of silanes depends on surface hydroxylation and various preparation methods of the colloidal probe surfaces are reported in literature like probe cleaning with different solvents, UV cleaning and plasma cleaning summarized in [18]. Additionally, plasma cleaning can be further optimized with the selection of gases, like plasma activation with H_2_, as reported by [24]. Multiple authors reported the gas phase deposition of organosilane monolayers [[25], [26], [27], [28], [29], [30]]. Hozumi et al. first demonstrated the preparation of fluoroalkyl silane monolayers under atmospheric pressure at 100 °C–150 °C reaching their final film thicknesses after one hour. The main advantage compared to solvent based deposition of organosilanes for colloidal probe preparation is the lack of the solvent in terms of an additional source of contamination, unwanted solvent glue interactions and the large amounts of solvents needed for sample or probe rinsing after the deposition. In the context of nanoimprint lithography Jung et al. reported fewer and smaller aggregates of the silane molecules on the surface, comparing vapor phase deposition and solvent based deposition of the silane [27]. Additionally, the authors suggested cycles of silane and water addition during monolayer deposition to increase film thickness and water contact angle by filling pinholes in the monolayer [27], while not being specific on the increase in contact angle and film thickness in the context of structural effects of silane aggregates. The addition of H_2_O in a curing step for the monolayer is adapted in the proposed procedure.

## Materials and methods

This section contains the used materials and methods including chemicals, probes, probe preparation and attachment procedures as well as the cantilever recycling procedure. As a general, remark the authors suggest for each surface described and handled in the procedure the use of powder free gloves.

### Chemicals

The chemicals used in this paper are summarized in Table 1.

**Table 1:** Used chemicals.

Name	Supplier
DYNASYLAN® F8261 (Tridecafluorotriethoxysilane)	Evonik Industries AG
OP-S 0.04 μm	Struers GmbH
Ethanol (ROTISOLV® HPLC Gradient Grade)	Carl Roth GmbH + Co. KG
Ber-Fix® Gel (UV glue)	Ber-Fix Klebstoffprodukte G.Häring & Ch.Franke GbR
Aerosol® 22	Sigma-Aldrich
KCl	Carl Roth GmbH + Co. KG
HCl	Carl Roth GmbH + Co. KG

### Probes and probe preparation

The colloids used are 19.59 μm (SD 0.69 μm) diameter SiO_2_ spheres supplied by microparticles GmbH. In order to remove reagents used in the manufacturing process the colloids were dispersed in ethanol (ROTISOLV® HPLC Gradient Grade) and treated 10 times in an ultrasonic bath for 10 min with a subsequent step of sedimentation, removal and re-addition of ethanol.

### Contact angle measurements and sample preparation

In order to assess the deposition of DYNASYLAN® F8261 on a hydroxylated sample surface, contact angle measurements were carried out with a DataPhysics OCA 50 on a quartz crystal before and after the functionalization with varying functionalization times, ranging from 0 min to 120 min.

To prepare the planar sample for functionalization, a clear quartz crystal was embedded in epoxy resin and gradually machine polished to a surface roughness of 0.3/0.5 nm (R_a_/R_q_). For each contact angle measurement the sample was re-polished with 0.04 μm amorphous SiO_2_ dispersion on an OP-Chem (Struers GmbH) polishing cloth for 1 min. The polishing was followed by 5 min of sonication in DI water. After sonication, the sample was rinsed with ethanol, swiped with a lint free cloth and sonicated again for 5 min in DI water to remove residual ethanol. Finally, the sample was dried with compressed air and plasma cleaned in O_2_ environment for 10 min.

After each contact angle measurement the sample was re-polished and cleaned as described above. Each singular conditioning step was at least measured six times with three repetitions in total, resulting in 18 data points per conditioning step. The measurements were performed with 0.3 μl DI water in a saturated environment.

### Probe attachment and functionalization procedure

A Park Systems XE100 atomic force microscope (AFM) was utilized for probe preparation, determination of the cantilever resonance frequency as well as topography and force measurements. The cantilevers used in this article are All-In-One B cantilevers supplied by nano and more GmbH and the colloids attached are 19.59 μm (SD 0.69 μm) diameter SiO_2_ spheres by microparticles GmbH, pre cleaned as described above and plasma cleaned in O_2_ environment in advance to the attachment to the cantilever. Prior to the attachment, the particles were deposited on a polished and cleaned surface similar to the sample preparation procedure suggested above, as standard microscope slides and cover slips are a possible source of surface contaminants, when directly in contact with the colloidal probe. After cantilever calibration as described in [18] the UV glue (Ber-Fix® UV Gel) was deposited on a substrate (e.g. cover slip). The glue was mechanically spread with a second cover slip to form a thin layer. In this application the use of cover slips is unproblematic as they are only in contact with the glue. Prior to the probe attachment the bottom of the cantilever end was covered with glue by slightly contacting the glue film with the cantilever by means of the atomic force microscope drives. In the case of an excessive amount of glue transferred to the cantilever, the glue can either be removed by solvents (acetone or ethanol) or plasma cleaning. The SiO_2_ particle is attached by moving the cantilever towards the particle by means of the AFM under guidance of an optical microscope and approaching the particle with the cantilever. After the attachment the cantilever is transferred to a microscope slide. The glue is set by a UV curing step by placing the cantilever and the microscope slide for 90 s directly below a UV-LED (wavelength 395 nm) setup, followed by a final plasma cleaning step for 10 min in O_2_ environment, ensuring a surface activation but not significantly affecting the cohesive and adhesive strength of the gule. In order to assess a surface contamination by glue in the probe attachment procedure the probe can either be topographically investigated by means of a standard scanning application with the AFM or by SEM imaging. Typically, no glue is visible on the top surface of the colloidal probe (see Fig. 1), as it forms a capillary with the lower half of the probe particle and the cantilever contact area.

**Fig. 2:**
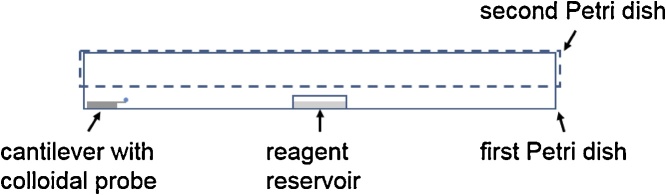
Functionalization configuration for cantilevers, the reagent reservoir, and Petri dishes.

Initially, the surface functionalization with DYNASYLAN® F8261 was reported in the temperature range from 100 to 150 °C by [26]. The Ber-Fix® Gel glue is temperature stable up to 120 °C according to the product specifications. Therefore, the functionalization of the probe was carried out at 115 °C in a drying cabinet. The temperature of 115 °C was chosen to compensate for the heat loss during loading of the drying cabinet and to avoid a drop below 100 °C. Additionally, a temperature below 120 °C acts as a safety precaution for a possible overshoot of the heat regulation circuit compensating for the heat loss. The cantilevers were placed in a Petri dish containing a reagent reservoir and were transferred to the drying cabinet. Initially, 50 μl of the silane was added to the reservoir and the setup was covered with a second Petri dish. The functionalization was carried out for 2 h with an additional curing step of 20 min with 100 μl water added to the reagent reservoir after the evaporation of the silane. The water curing step was adapted from [27], resulting in a total functionalization time of 140 min. The configuration of the components is depicted in Fig. 2. The authors like to emphasize that the Petri dishes do not seal the setup air tight, as this lead to unwanted effects in surface modification in previous tests.

### Cantilever recycling procedure

To detach the probe particle from the cantilever after contamination or degradation of its functionalization layer, the glue is removed by a plasma cleaning procedure. As demonstrated by Rudolph and Peuker [7] the change in resonance frequency can be utilized to calculate the mass of glue at the tip of a cantilever fixating the colloid in place, summarized in the following equations.

(1) $$m_{colloid+glue}=\frac{k}{4*\;\pi^{2}}*\left(\frac{1}{\omega_{attached}^{2}}-\frac{1}{\omega_{initial}^{2}}\right)$$

(2) $$m_{colloid}=\frac{4}{3}*\pi *R^{3}*\rho_{colloid}$$

**Fig. 3:**
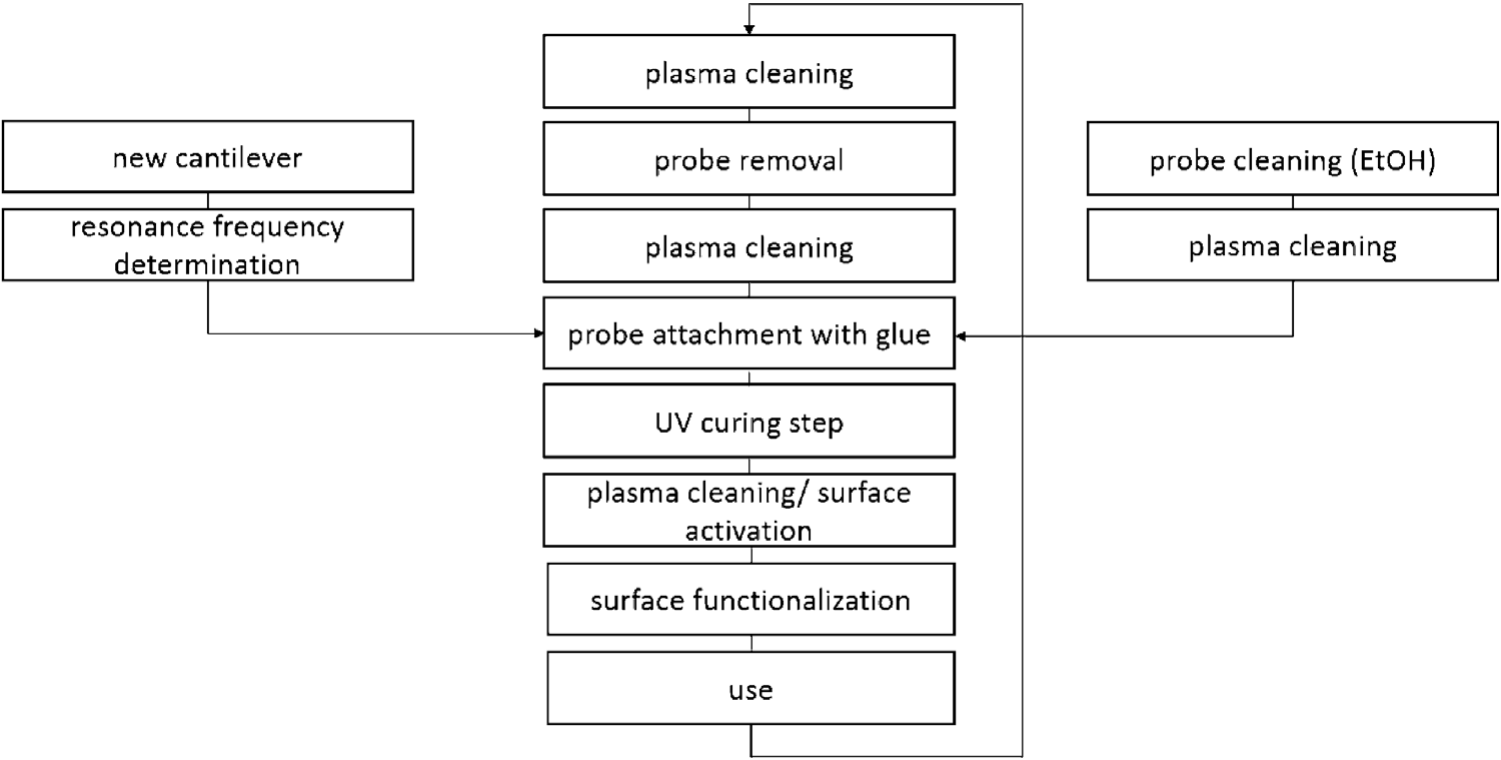
Probe preparation, functionalization and recycling procedure.

The change in glue mass is calculated combining Eqs. (1) and (2) for different times during the plasma cleaning procedure and is used to assess the glue mass retention over time, giving an operating window for probe functionalization, utilization and removal of the probe particle. After the plasma cleaning procedure, the probe can be removed from the cantilever with water, ethanol or acetone or by aid of micromanipulation, which is preferable to avoid possible contamination of the reflective coating by a rinsing step. The glue removal by the plasma cleaning procedure is favorable in comparison to solvent or acid based removal strategies in terms of additional contaminants either of particular or liquid kind and stability of the reflex coating of the used cantilevers. The probe removal and attachment cycle is given in Fig. 3, including a final plasma cleaning step to remove residual glue in the probe-cantilever contact area, before reattachment of a pristine probe particle. In order to assess possible changes in mechanical properties of a cantilever in the plasma cleaning procedure two pristine cantilevers were treated in O_2_ plasma for 10 h with periodic determination of the resonance frequency.

### Adhesion measurements

To approve the applicability of the proposed procedure for its intended use, CP-AFM measurements on mineral surfaces are carried out with one cantilever and three different colloidal probes on a SnO_2_ sample functionalized with Aerosol22 in 60 ml 10^−2^ mol/l KCl solution set to pH 3 by HCl, resulting in a contact angle of about 89°. The measurements were intendedly not carried out on the SiO_2_ contact angle proxy sample as a contact angle of about 107° does not reflect a realistic case in the context of the intended use of the colloidal probes. The force limit was set to 75 nN and an area of 90 × 90 μm was mapped with 16 × 16 points. The same area was repeatedly mapped to assess the change of adhesion between the sample surface and the probe of the points measured.

## Method validation

This section shows the results of the proxy contact angle measurements and the results of the plasma cleaning procedure as well as CP-AFM adhesion measurements.

**Fig. 4:**
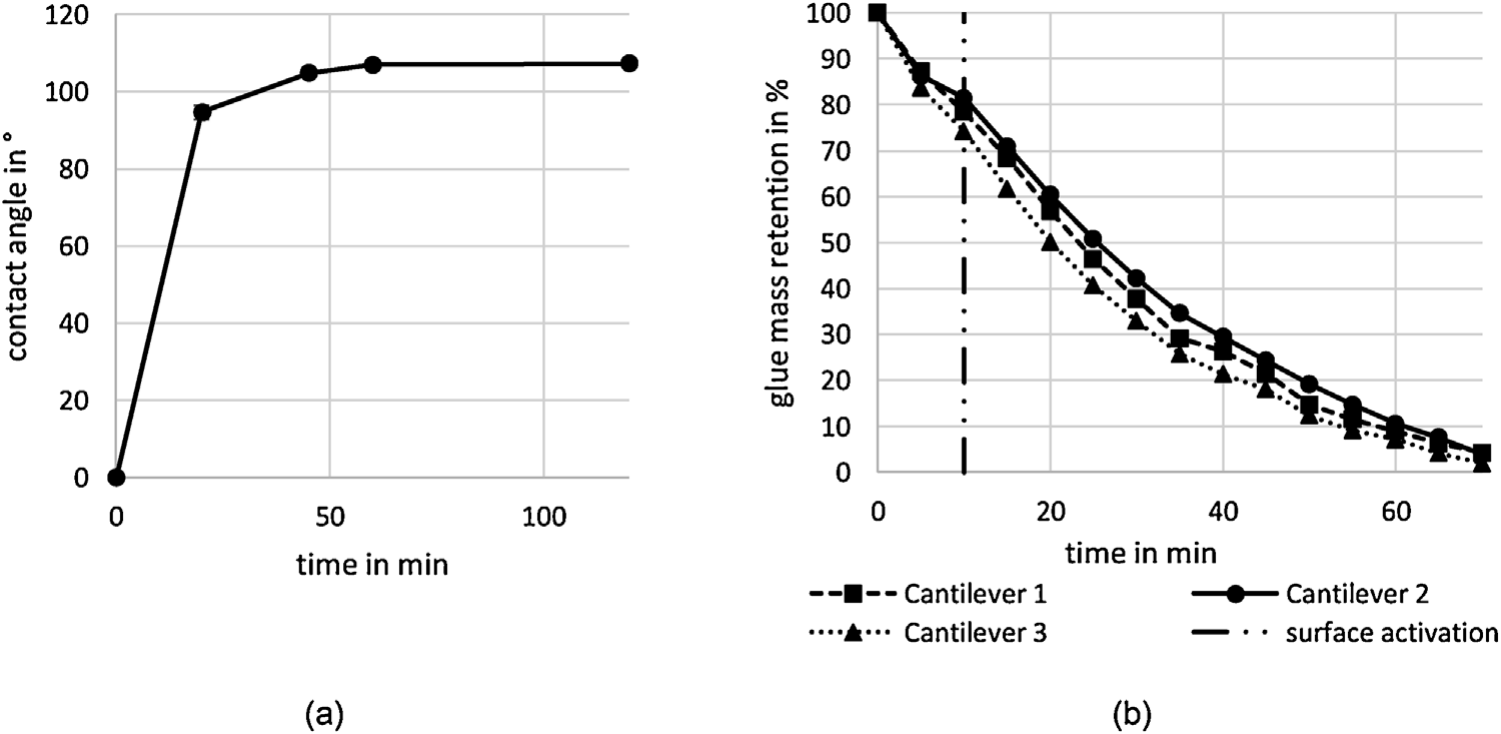
Results of the surface modification, i.e. contact angle of the sample surface as a function of functionalization time (**a**) and plasma cleaning procedure, i.e. residual mass of glue on a cantilever as a function of plasma cleaning time (**b**).

Fig. 4 shows the contact angle of the SiO_2_ sample with varying deposition times at 115 °C with the standard deviation (a), indicating that the contact angle forms a plateau after 45 min. and is reproducible. In panel (b) the results of the glue removal procedure are displayed as the glue mass retention over time. As mentioned in the probe attachment and functionalization procedure prior to the functionalization 10 min of plasma cleaning were used as a surface cleaning and activation measure. Within these 10 min about 20% of the glue mass is removed from the cantilever. Empirically, this value is still suitable for a stable fixation of the colloidal probe, thus giving an operation window for the cantilever with a surface hydroxylation and functionalization (10 min) as well as the utilization and removal phase (well above 10 min). This removal procedure can be optimized by an intensified plasma cleaning with a higher power output of the cleaner and a further reduction in the total functionalization times, if needed. The long-term plasma treatment for the cantilevers without attached glue or particles resulted in a 0.09% average change of the resonance frequency. Considering the deviation in spring constant calibration, probe diameter and cantilever dimensions determination as well as the degree of variation of surface hydrophobicity of the probes this change seems to be negligible.

**Fig. 5:**
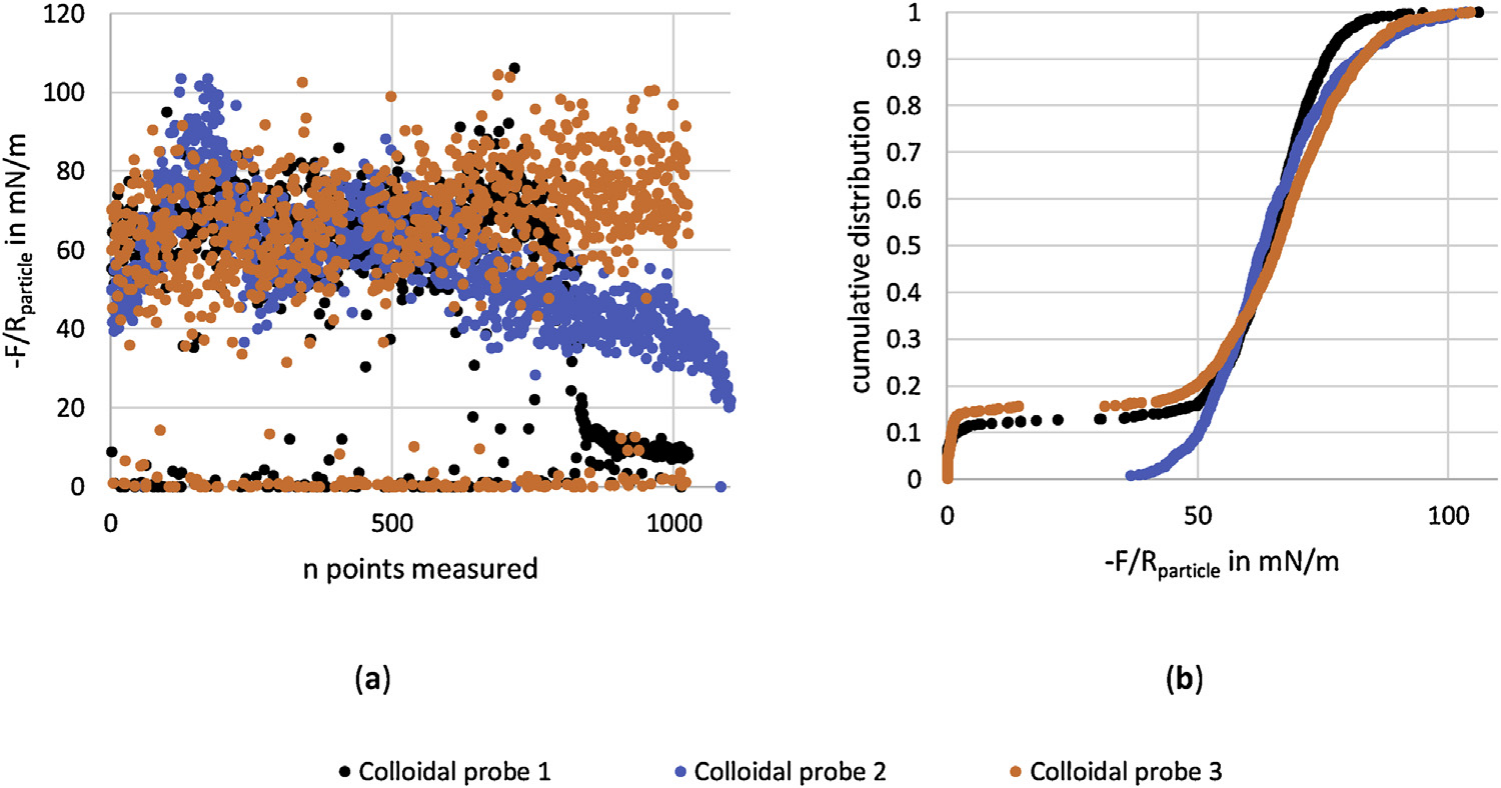
Exemplary force measurement with one cantilever and three subsequent utilized colloidal probes, including durability measurements (**a**) and resulting force distributions (**b**).

Fig. 5 displays the adhesion measurements with one cantilever and three subsequently used colloids on a functionalized SnO_2_ sample. The results are given as a total number of points measured (a) and as cumulative force distributions (b) of the three colloids used. The total number of points measureable without significant drop off in adhesion force are 803, 668 and 1023 point of colloid 1 to 3 respectively. The cumulative distribution are constructed including those threshold values and display a good comparability between the colloids used down to the 20% cumulative values. Differences below 50 mN/m detachment force in the cumulative plot between colloidal probe 1 and 3 must stem from an uneven surface functionalization of the underlying sample, as these measurements reflect zero adhesion in between points with increased adhesion force measured, thus not being related to a lack in surface hydrophobization of the colloid.

## Conclusion

In this method paper, we presented a protocol to efficiently prepare hydrophobic colloidal probes, which substantially reduces the time for probe preparation and allows a recycling of cantilevers in case of probe particle contamination. The contaminated probe can be detached from the cantilever by a plasma cleaning procedure and the cantilever can be reequipped with a pristine colloidal probe, minimizing the time for glue setting and probe functionalization. The proposed procedure avoids the standard cleaning procedures with their limited use for particulate contaminations and allows the reuse of costly cantilevers with abundantly available colloids, allowing more extensive investigations on samples, which are likely to cause probe contamination. Additionally, new opportunities arise from interchangeable colloidal probes such as investigations with irregularly shaped particles in interfaces, allowing statistically relevant observations with a single cantilever also reducing the deviations caused by cantilever calibration.

## Funding

This research was funded by the POF III (program-oriented funding) of the Helmholtz Association designated for flotation research in the EMR program under subtopic 5 (resource technologies).

## Conflict of interest

The authors declare no conflict of interest.
